# Identification and validation of a T-cell-related MIR600HG/hsa-mir-21-5p competing endogenous RNA network in tuberculosis activation based on integrated bioinformatics approaches

**DOI:** 10.3389/fgene.2022.979213

**Published:** 2022-09-20

**Authors:** Guo-Hu Hong, Qing Guan, Hong Peng, Xin-Hua Luo, Qing Mao

**Affiliations:** ^1^ Department of Infectious Disease, Guizhou Provincial People’s Hospital, Guiyang, China; ^2^ Department of Dermatology, The First People’s Hospital of Guiyang, Guiyang, China; ^3^ Department of Infectious Disease, The First Hospital Affiliated to Army Medical University, Chongqing, China

**Keywords:** active tuberculosis, disease classification, CD4+ T cell, adaptive immunity, competing endogenous RNA network

## Abstract

**Background:** T cells play critical roles in the progression of tuberculosis (TB); however, knowledge regarding these molecular mechanisms remains inadequate. This study constructed a critical ceRNA network was constructed to identify the potentially important role of TB activation *via* T-cell regulation.

**Methods:** We performed integrated bioinformatics analysis in a randomly selected training set from the GSE37250 dataset. After estimating the abundance of 18 types of T cells using ImmuCellAI, critical T-cell subsets were determined by their diagnostic accuracy in distinguishing active from latent TB. We then identified the critical genes associated with T-cell subsets in TB activation through co-expression analysis and PPI network prediction. Then, the ceRNA network was constructed based on RNA complementarity detection on the DIANA-LncBase and mirDIP platform. The gene biomarkers included in the ceRNA network were lncRNA, miRNA, and targeting mRNA. We then applied an elastic net regression model to develop a diagnostic classifier to assess the significance of the gene biomarkers in clinical applications. Internal and external validations were performed to assess the repeatability and generalizability.

**Results:** We identified CD4^+^ T, Tr1, nTreg, iTreg, and Tfh as T cells critical for TB activation. A ceRNA network mediated by the MIR600HG/hsa-mir-21-5p axis was constructed, in which the significant gene cluster regulated the critical T subsets in TB activation. MIR600HG, hsa-mir-21-5p, and five targeting mRNAs (BCL11B, ETS1, EPHA4, KLF12, and KMT2A) were identified as gene biomarkers. The elastic net diagnostic classifier accurately distinguished active TB from latent. The validation analysis confirmed that our findings had high generalizability in different host background cases.

**Conclusion:** The findings of this study provided novel insight into the underlying mechanisms of TB activation and identifying prospective biomarkers for clinical applications.

## Introduction

Tuberculosis (TB) is a highly contagious respiratory disease caused by *Mycobacterium tuberculosis* (*M. tb*) infection that poses a severe threat to public health globally (Cohen et al., 2019). The interaction between pathogenic virulence and organismal immunity largely dictates TB progression. After *M. tb* infection, a series of complex immune processes, most cases enter a latent state through the long-term co-existence with the pathogens. *M. tb* invasion and consequent organism damage initiate inflammatory responses in cases of immune dysfunction, leading the latent state to progress to an active state. Globally, around 5%–15% of latent TB cases eventually progress to active disease (Luo et al., 2019), contributing to one of the leading causes of death worldwide (World Health Organization, 2021). The in-depth investigation of the underlying mechanisms of TB progression has been a research priority in recent decades.

The adaptive immune responses mediated by T cells play a crucial role in the equilibrium of the host against *M. tb* (Jasenosky et al., 2015; Chai et al., 2020); these responses include antigen recognition, inflammatory homeostasis maintenance, and granuloma formation. Several T-cell biological processes, such as development, differentiation, and sensitization, are involved in suppressing TB activity (Feruglio et al., 2015; Huang et al., 2019). In recent years, studies based on cohort microarray data analysis have identified T-cell-related mRNAs, microRNAs (miRNAs), and long non-coding RNAs (lncRNAs) as critical biomarkers in distinguishing active from latent states (Walzl et al., 2018; Sinigaglia et al., 2020; Kundu and Basu, 2021). However, most of these markers were identified *via* data-driven strategies; thus, the regulating mechanisms of these genes in TB progression remain under-characterized. The results of the bioinformatics approaches applied in the present study identified a lncRNA/miRNA axis-mediated competing endogenous RNA (ceRNA) network that is involved in TB activation through T-cell regulation.

**FIGURE 1:**
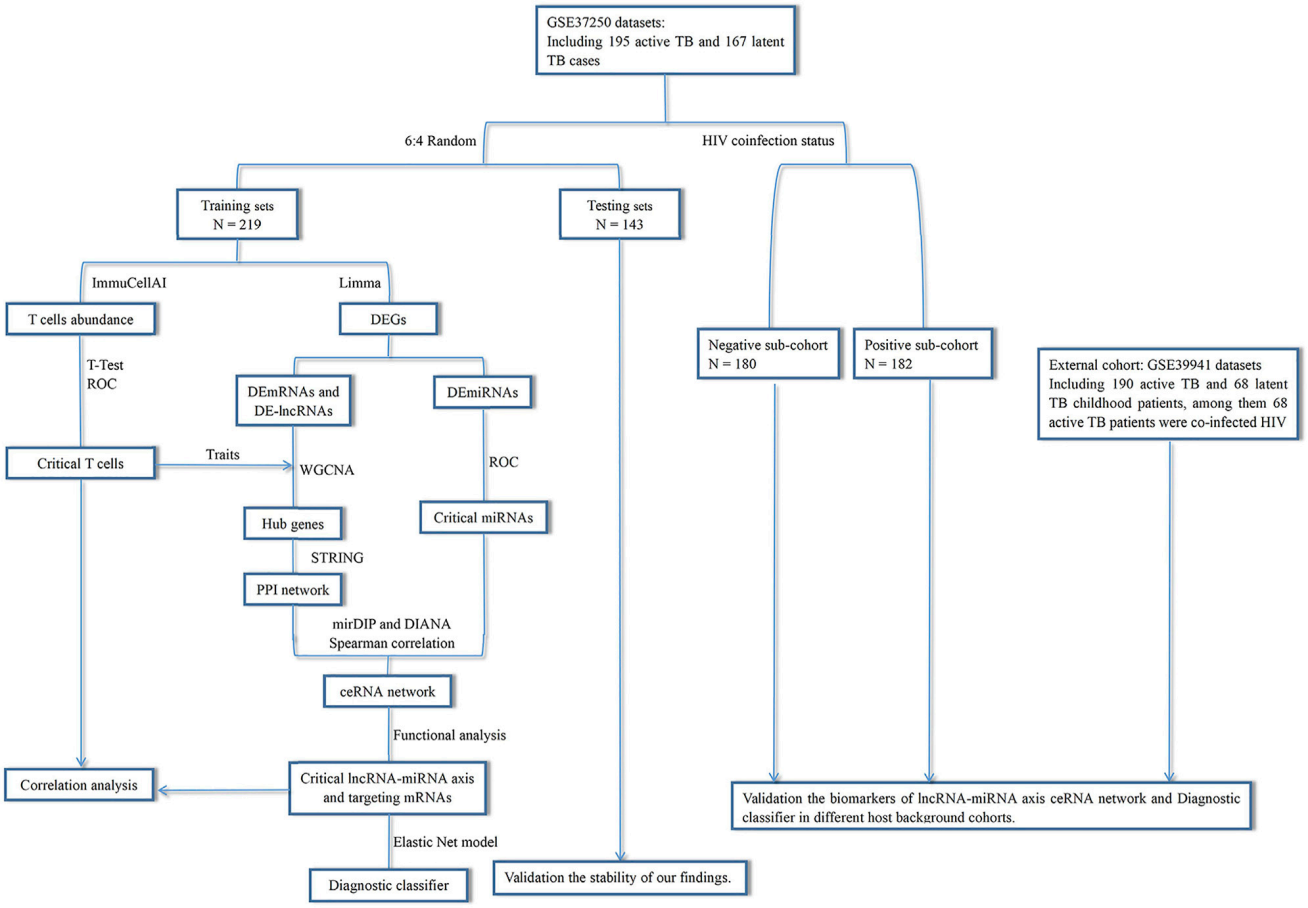
Overview of the study design.

**FIGURE 2:**
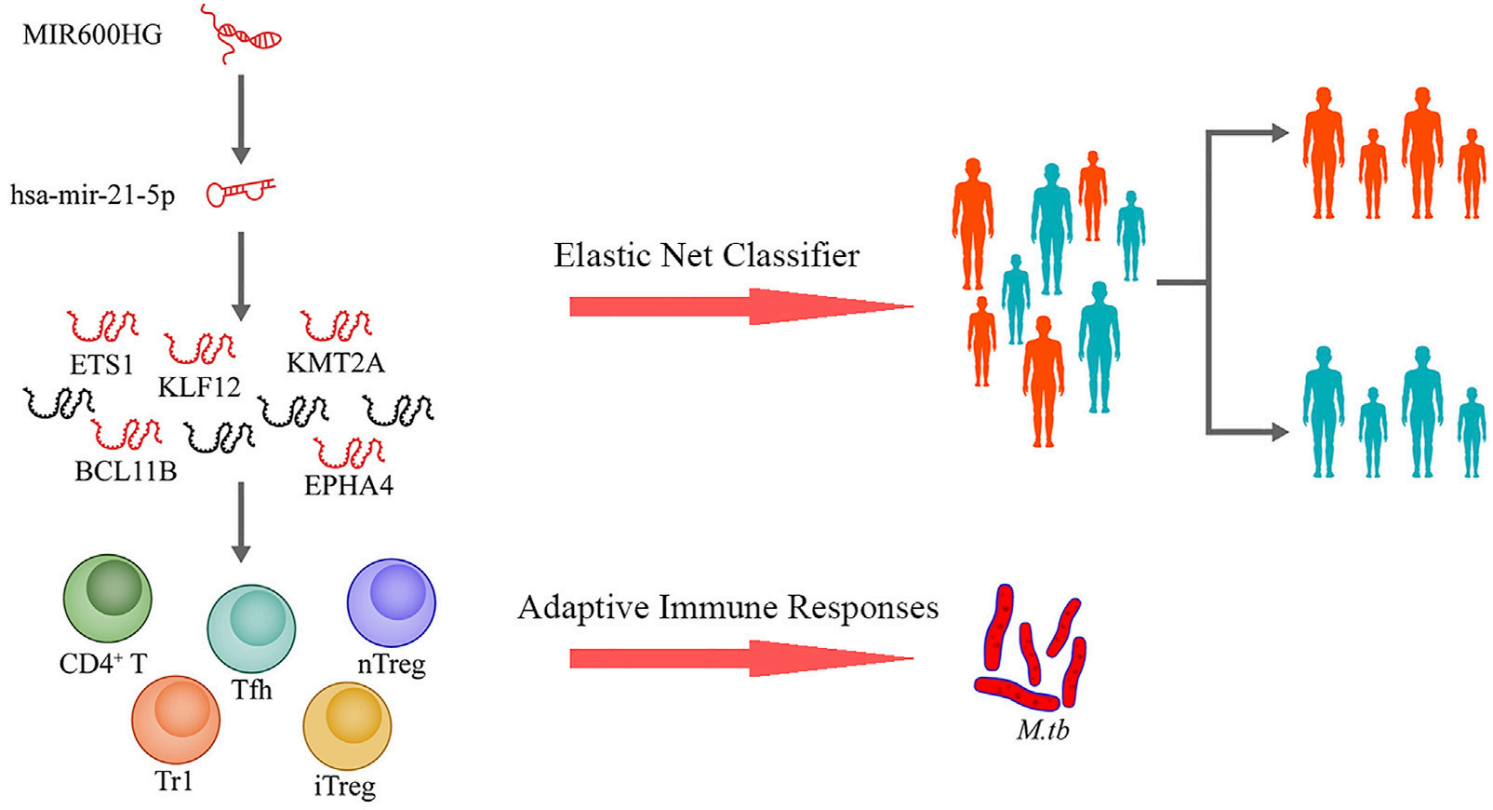
Proposed scheme for this study.

This study was designed as shown in Figure 1. We used TB samples from the GSE37250 dataset (Kaforou et al., 2013) and randomly selected a portion of them for data mining. Using integrated bioinformatics and statistical approaches, we constructed a ceRNA network mediated by MIR600HG/hsa-mir-21-5p axis that may play an important role in TB activation by regulating some CD4^+^ T subsets. To assess the clinical significance of the regulatory genes in this ceRNA network, we employed an elastic net regression model (Friedman et al., 2010) to construct a diagnostic classifier based on MIR600HG, hsa-mir-21-5p, and the targeting mRNAs. Internal validation confirmed the repeatability and evaluated the generalizability of these markers in HIV-negative and HIV-positive sub-groups of patients. As the external validation should be strictly constrained by the target population size and implementation criterion, we used independent datasets with similar scales and execution strategies to perform this assessment. The results of external validation showed the high applicability of these markers in pediatric cases. The results of this study extend our understanding of the immunological mechanisms involved in TB activation and provide novel potential diagnostic biomarkers for clinical applications (Figure 2).

## Materials and methods

### Data resources

We obtained the GSE37250 dataset from the Gene Expression Omnibus (GEO) database. This adult cohort contains 195 active and 167 latent cases of TB in patients from South Africa and Malawi. These cases were randomly separated into training and testing sets at a ratio of 6:4. The training sets contained 116 active and 103 latent cases; among them, 60 active and 53 latent cases were co-infected with HIV. The testing sets contained 79 active and 64 latent cases; among them, 38 active and 31 latent cases were co-infected with HIV. The GSE39941 (Anderson et al., 2014) dataset was also obtained for use as the external validation cohort. This pediatric cohort contains 190 active and 68 latent cases from Africa; among them, 68 active cases were co-infected with HIV, with similar proportions of the target population and TB status diagnostic criteria as those in the GSE37250 dataset. Before performing the bioinformatics and statistical operations in this study, all microarray expression data were transformed using the base-2 logarithm through normalized data.

### Identification of critical T cells

The abundance of 18 types of T cells for each case was computed using the Immune Cell Abundance Identifier (ImmuCellAI (Miao et al., 2020)) platform. After comparing the differences in T-cell abundance between active and latent cases, T cells with significant differences were assessed by receiver operating characteristic (ROC) curve analysis. Based on a threshold of the area under the curve (AUC) of < 0.70, we identified critical T-cell types after excluding those that showed poor performance in classification.

### Screening differentially expressed genes and identification of candidate DEmiRNA

The fold-changes (FCs) of each probe in the training sets were calculated after adjusting the false discovery rate (FDR) using the Benjamini–Hochberg (BH) algorithm. The differentially-expressed genes (DEGs) were then filtered out as criteria based on an adjusted *p*-value <0.05 and |FC| > 1.5. DEmiRNAs, DElncRNAs, and DEmRNAs were categorized according to the GENCODE release 35 annotations. ROC curves were used to identify candidate DEmiRNAs to eliminate those probes with an AUC value of <0.7.

### Co-expression analysis of differentially expressed genes

The DElncRNA and DEmRNA expression data were included in the gene matrix, while the TB states and critical T-cell abundances were included as the clinical traits. The weighted gene co-expression network analysis (WGCNA) (Langfelder and Horvath, 2008) was used to identify the gene module associated with the clinical traits. After discarding outlier cases, a hierarchical clustering tree was constructed based on the topological overlap matrix dissimilarity measure, while the soft-thresholding power was set as the scale-free R^2^ accumulated up to 0.8. After merging the similar modules at a threshold of 0.25, the correlations between each module and clinical trait were calculated to identify the most significant module. The module membership (MM) represented the relationship between genes in a given module, while the gene significance (GS) defined the correlation between each gene and the clinical traits. The hub gene in the significant module showed an MM of >0.7 and a GS of >0.3.

### Construction of a competing endogenous RNA regulatory network

The DIANA-LncBase v3 tool (Karagkouni et al., 2020) was used to predict hub DElncRNAs targeting candidate DEmiRNAs in the reverse direction. A protein-protein interaction (PPI) network of hub mRNA was constructed using the STRING (Szklarczyk et al., 2021) database and visualized in Cytoscape. In the PPI network, the targeting mRNAs of the critical miRNAs were determined using the microRNA Data Integration Portal (mirDIP) (Tokar et al., 2018) with the top 5% confidence class. The ceRNA regulatory network was obtained after expanding to the first-level neighbor nodes of the targeting mRNAs. We enriched for the Gene Ontology biological processes (GOBP) and Kyoto Encyclopedia of Genes and Genomes (KEGG) pathways in the PPI network and ceRNA regulatory gene clusters using the Metascape (Zhou et al., 2019) platform. The critical gene cluster was then identified according to the functional annotation. The resulting lncRNAs, miRNAs, and targeting mRNAs were identified as critical gene biomarkers in TB activation. The correlations between gene biomarkers and the critical T cells and between critical miRNAs and lncRNAs/mRNAs were confirmed *via* Spearman coefficient analyses.

### Clinical significance assessment and validation

The elastic net regression model with a parameter minimum of λ was used to develop a diagnostic classifier based on the gene biomarkers expression data after10-fold cross-validation. The classification accuracy in distinguishing active TB using the gene biomarkers and scoring system was assessed by ROC curve analysis. The results were validated in the testing sets to assess the repeatability of our findings. the validation analysis in the internal HIV-negative and HIV-positive sub-cohorts provided a better case for assessing the generalizability in cases with different immunological backgrounds. The validation results in the external cohort were used to assess the potential application of the classifier in pediatric cases.

### Statistical tools

R software was used to perform the statistical analysis, using packages including limma, ggpubr, WGCNA, pROC, caret, and glmnet. *P* < 0.05 was considered to indicate statistical significance.

## Results

### Critical T cells

**FIGURE 3:**
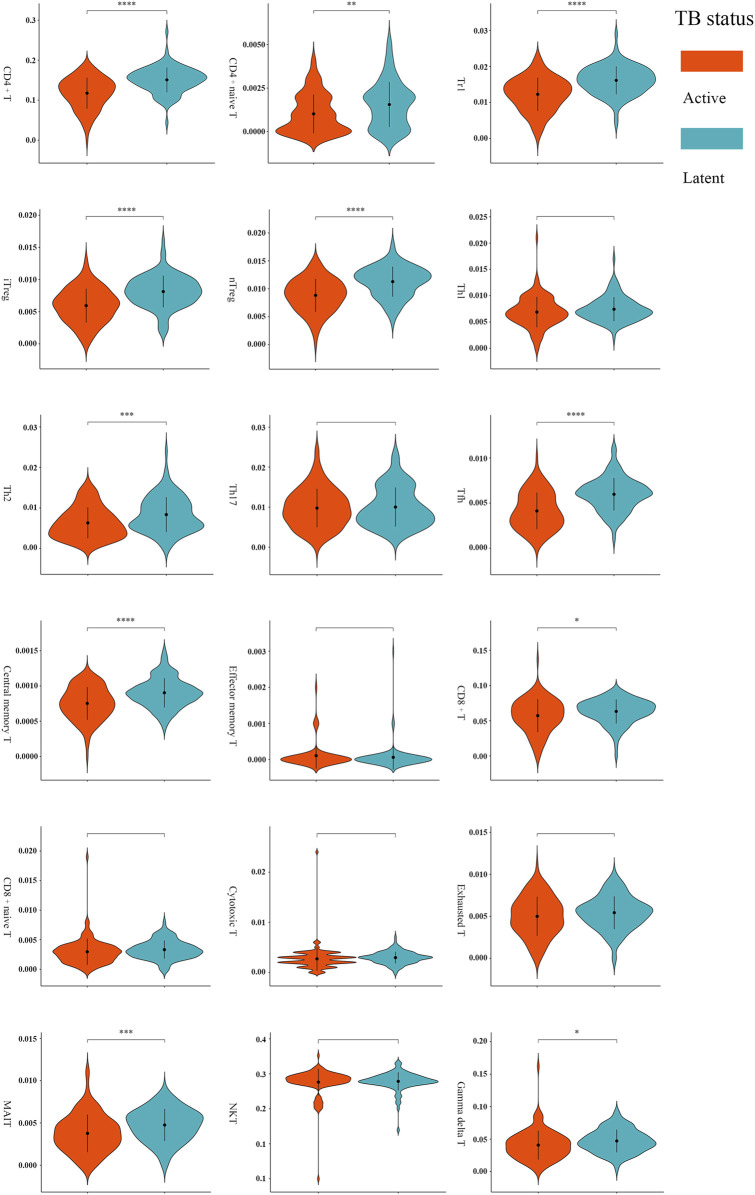
Comparisons of T-cell abundance between active and latent TB cases in the training set. *p < 0.05, **p < 0.01, ***p < 0.001, and ****p < 0.0001 by t-test.

A total of 11 types of immune cells showed significantly different abundance levels between active and latent cases (Figure 3). These types, including CD4^+^ T, CD8^+^ T, naive CD4^+^ T, type 1 regulatory T (Tr1), natural regulatory T (nTreg), inducible regulatory T (iTreg), T helper 2 (Th2), follicular helper T cell (Tfh), central memory T (Tcm), γδ T, and mucosal-associated invariant T (MAIT), showed significantly lower abundances in active cases. After excluding the poorly discriminating types based on an AUC threshold of <0.70, CD4^+^ T, Tr1, nTreg, iTreg, and Tfh were identified as the critical T cells, with AUCs of 0.75 [95% CI, 0.68–0.81], 0.74 [95% CI, 0.67–0.80], 0.73 [95% CI, 0.66–0.80], 0.73 [95% CI, 0.66–0.80], and 0.75 [95% CI, 0.68–0.81], respectively (Supplementary Table S1).

### Identification of differentially expressed genes and candidate DEmiRNA

Comparisons of active and latent TB cases revealed 77 lncRNAs, 17 miRNAs, and 1806 mRNAs as significant. Among these DEGs, 34 lncRNAs, 10 miRNAs, and 980 mRNAs were up-regulated in active cases. The others were down-regulated (Supplementary Figure S1). The ROC analysis revealed two miRNAs as candidate DEmiRNAs (Supplementary Table S2): hsa-mir-21-5p was up-regulated in active cases, with an AUC of 0.84 [95% CI, 0.79–0.90], while hsa-mir-339-5p was down-regulated in active cases, with an AUC of 0.77 [95% CI, 0.71–0.83].

### Co-expression analysis and the identification of hub differentially expressed genes

**FIGURE 4:**
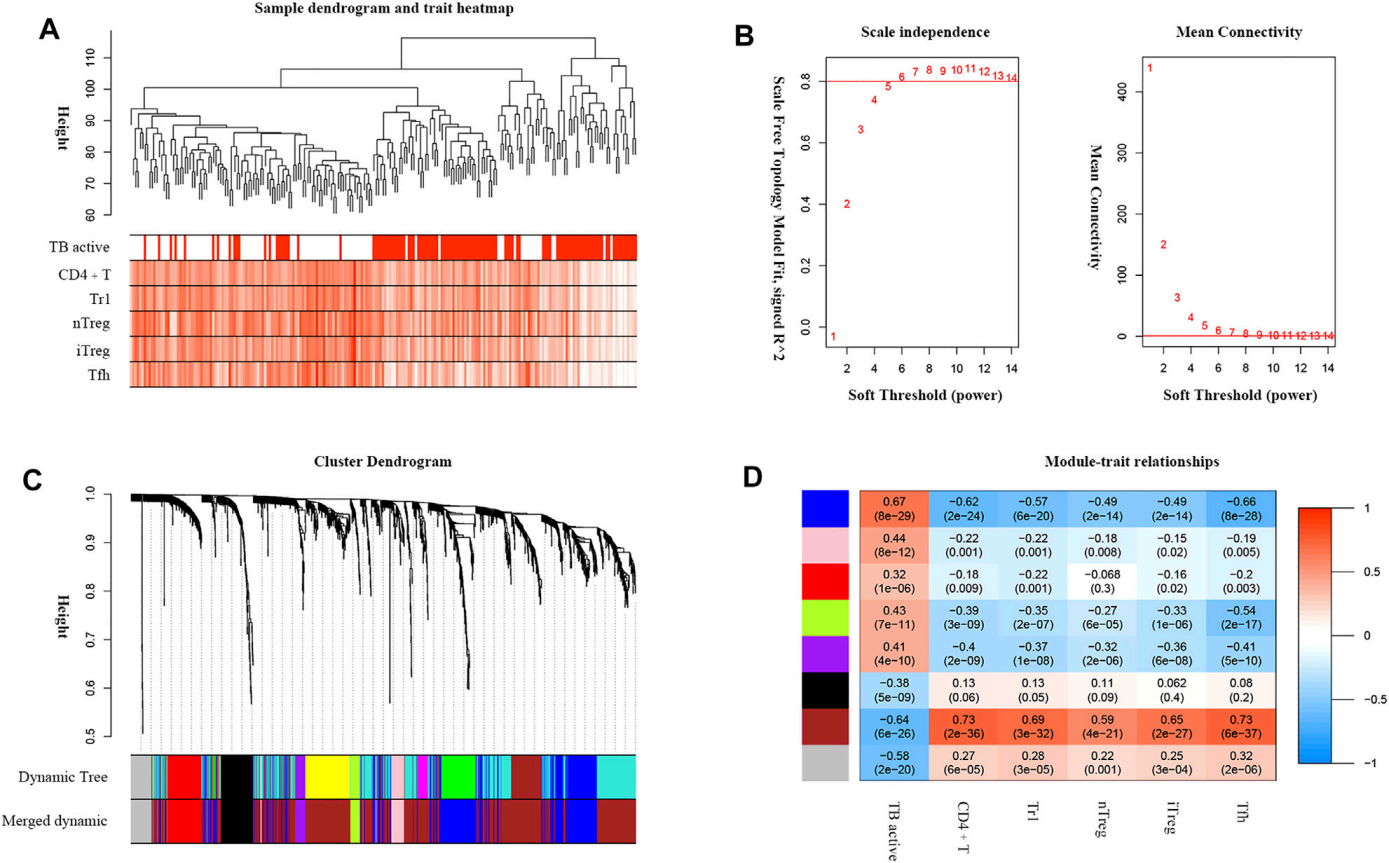
WGCNA analysis of DEGs associated with TB active status and critical T-cell abundance. **(A)** TB active status and T-cell abundance of samples clustered according to DEG expression. **(B)** Analysis of the network topology for various soft-threshold powers. The auxiliary line on the longitudinal axis in the scale-free R^2^ shows a value of 0.8. **(C)** Dendrogram of DEGs clustered based on the measurement of dissimilarity (1-TOM). **(D)** Heatmap of the correlations between the module eigengenes and the clinical traits. The numbers represent the correlation coefficients, with the corresponding *p* values indicated in brackets.

TB progression and critical T-cell abundance were well clustered by the average linkage method based on the DEG expression data (Figure 4A). A scale-free network was constructed with a soft-thresholding power *β* = 6 (Figure 4B). After excluding the unclustered gray eigengenes, 11 modules were obtained using the dynamic tree algorithm. After merging the similar types, seven modules were included in the calculations of their correlations with clinical traits (Figure 4C). The brown module showed the strongest negative relationship with active TB and positive relationship with critical T-cell abundance (Figure 4D); therefore, it was identified as the significant module. We screened 197 genes in the brown module as potential hub genes, including two lncRNAs (MIR600HG, PP7080) and 195 mRNAs.

### Construction of the competing endogenous RNA regulatory network and functional enrichment analysis

**FIGURE 5:**
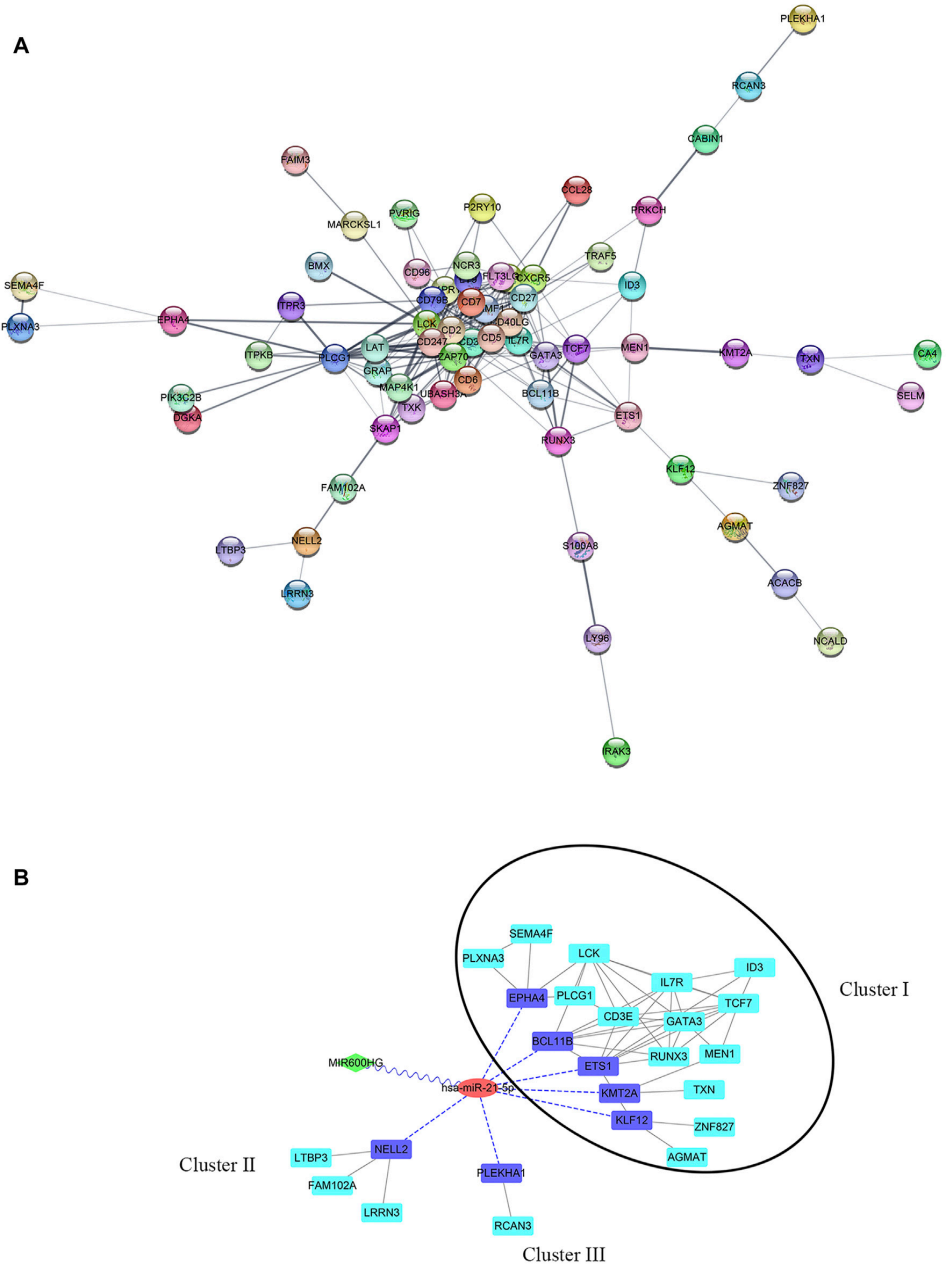
**(A)** PPI network of hub genes in the brown module. **(B)** ceRNA network. The green diamond node represents lncRNA, the red circular node represents miRNA, the blue square nodes represented the targeting mRNAs of hsa-mir-21-5p, and the turquoise square nodes represent the first-level neighbors of the targeting mRNA. The grey edges indicate interactions between proteins. The blue dotted edges indicate targeting from miRNA to mRNAs. The wavy edges indicate targeting from lncRNA to miRNA. The black circular frame indicates the major gene cluster in the ceRNA network.

By predicting the interaction relationships between each hub mRNA in the STRING database, a PPI network containing 68 genes was obtained (Figure 5A). MIR600HG and PP7080 were identified as the hub lncRNAs in the brown module and were down-regulated in active cases. Due to the competitive principle in ceRNA networks, up-regulated miRNAs could be considered potential targets; therefore, hsa-mir-21-5p was identified. Using the DIANA-LncBase tool, only MIR600HG was predicted as the targeting lncRNA of hsa-mir-21-5p, with a significant negative correlation between them (Spearman R = −0.46, *p* < 0.01). The mirDIP filtered seven genes (BCL11B, ETS1, EPHA4, KLF12, KMT2A, PLEKHA1, and NELL2) in the PPI network as the targeting mRNAs of hsa-mir-21-5p. Reserving the first-level neighbor nodes of these targeting mRNAs, we obtained a ceRNA regulatory network containing three gene clusters (Figure 5B). The cluster I gene group clustered 19 genes together, including five hsa-mir-21-5p targeting mRNAs (BCL11B, ETS1, EPHA4, KLF12, and KMT2A).

**FIGURE 6:**
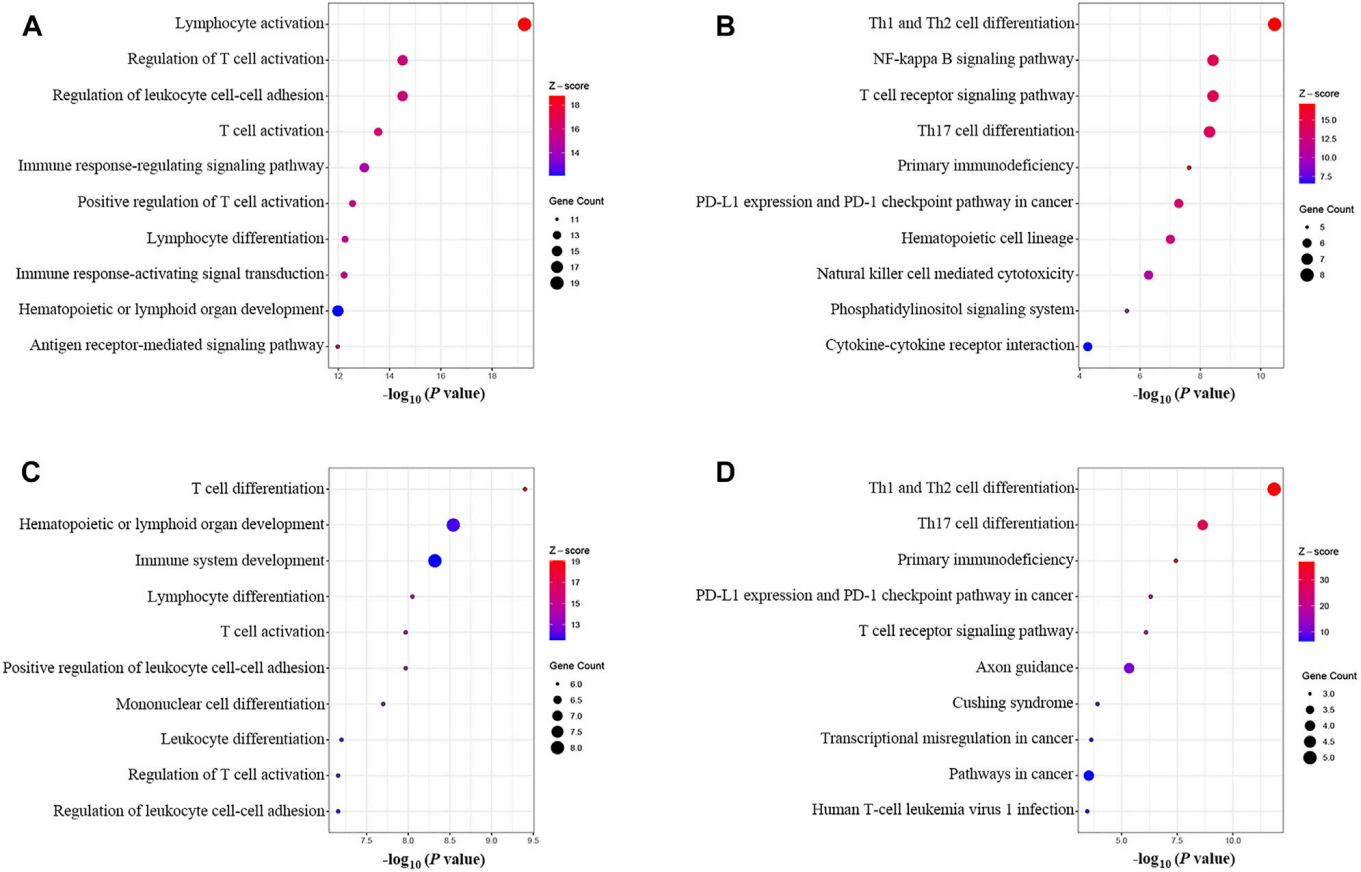
GOBP and KEGG enrichment analysis. **(A)** GOBP enrichment of the PPI network. **(B)** KEGG enrichment of the PPI network. **(C)** GOBP enrichment of the main gene cluster in the ceRNA network. **(D)** KEGG enrichment of the main gene cluster in the ceRNA network.

The results of the GOBP enrichment analysis revealed that T-cell-associated processes were the significant biological functions in the PPI network (Figure 6A). The KEGG enrichment analysis (Figure 6B) showed that these genes were involved in several immune-associated pathways, such as T-cell differentiation and NF-kappa B, and programmed cell death protein-1 (PD-1) checkpoints. The enrichment analysis of the cluster I gene group revealed high functional representativeness across the entire PPI network (Figures 6C,D). The results of the Spearman analysis confirmed significant negative correlations between hsa-mir-21-5p and BCL11B, ETS1, EPHA4, KLF12, and KMT2A (R = −0.62, −0.57, −0.39, −0.59, and −0.55, respectively, all *p* < 0.01).

### Clinical significance assessment

**FIGURE 7:**
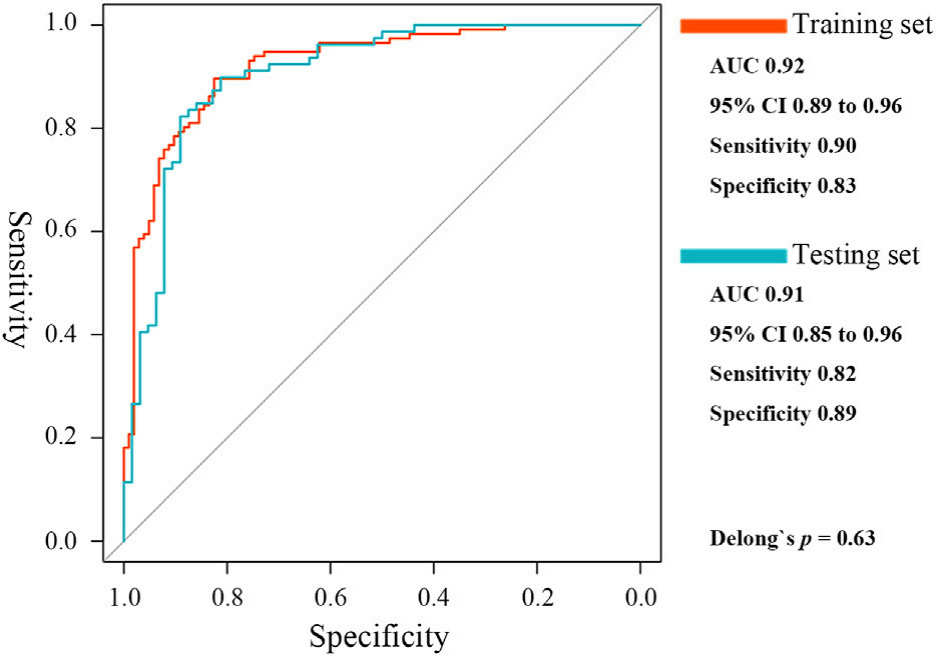
ROC curves of the elastic net diagnostic classifier in the training and testing sets.

A ceRNA network mediated by MIR600HG/hsa-mir-21-5p axis, which could be involved in TB activation, was constructed. BCL11B, ETS1, EPHA4, KLF12, and KMT2A were identified as the critical targeting mRNAs in this ceRNA network. Contrary to hsa-mir-21-5p, MIR600HG, BCL11B, ETS1, EPHA4, KLF12, and KMT2A were down-regulated in active cases and were considered credible diagnostic indicators with high accuracy (Supplementary Table S3). As the results of the Spearman analysis showed, the seven gene biomarkers were significantly correlated with the critical T-cell types (Supplementary Table S4). Therefore, these genes were used to fit the elastic net regression model with 10-fold cross-validation. Based on α = 0.1 and λ = 0.0124, a scoring classifier was determined, as follows: 1.07*hsa-mir-21-5p - 0.73*MIR600HG - 0.02*BCL11B–0.62*ETS1–0.40*EPHA4–0.04*KLF12–0.65*KMT2A. The scoring classifier provided excellent accuracy in distinguishing active from latent cases, with an AUC of 0.92 [95% CI, 0.89–0.96] (Figure 7).

### Validation analysis

In the testing set, hsa-mir-21-5p expression showed significant negative correlations with MIR600HG, BCL11B, ETS1, EPHA4, KLF12, and KMT2A (R = −0.47, −0.69, −0.62, −0.48, −0.69 and −0.67, respectively, all *p* < 0.01). The gene biomarkers significantly correlated with the critical T cells showed similar trends in the training set (Supplementary Table S4). CD4^+^ T, Tr1, nTreg, iTreg, and Tfh showed significantly lower levels of abundance in active compared to latent cases (Supplementary Figure S2). The results of the ROC curve analysis demonstrated the reasonable accuracy of all critical T cells in diagnosing TB progression (Supplementary Table S5). The elastic net scoring classifier showed comparable accuracies between the test and training sets (Figure 7). However, Delong’s test revealed no significant differences (*p* = 0.63). Each gene biomarker showed a similar expression trend and diagnostic accuracy to that of the training set (Supplementary Table S6). The high reproducibility and confidence were confirmed by validation in the testing set.

**FIGURE 8:**
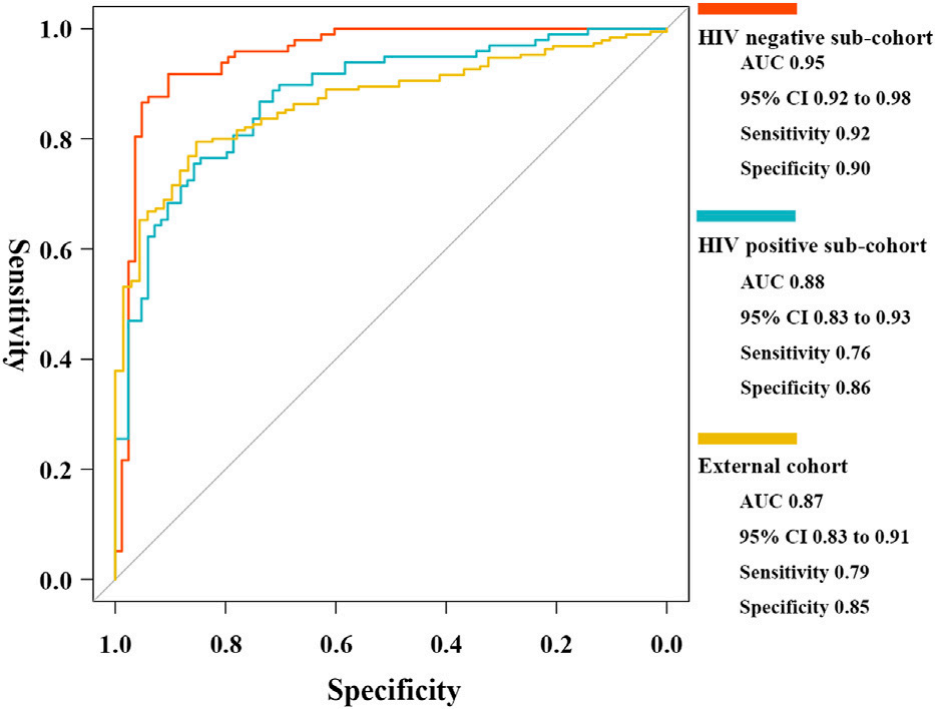
ROC curves of the elastic net diagnostic classifier in HIV-negative/positive sub-cohorts and the external cohort.

To assess accuracy in different host background cases, two internal sub-cohorts and one external independent cohort were used for validation. The five critical T-cell types showed lower abundance levels in active cases (Supplementary Figure S3), as well as reasonable diagnostic accuracy (Supplementary Table S7). The results of the ROC curve analysis indicated the high generalizability of the scoring classifier in cases of HIV-negative adults, HIV-positive adults, and pediatric cases, with AUCs of 0.95 [95% CI, 0.92–0.98], 0.88 [95% CI, 0.83–0.93], and 0.87 [95% CI, 0.83–0.91], respectively (Figure 8). In the three validation cohorts, every gene biomarker showed similar expression trends and diagnostic efficiencies, consistent with the training set (Supplementary Table S8). Based on the validated results, we believed that the ceRNA network, mediated by the MIR600HG/hsa-mir-21-5p axis, played an important role in cases with different backgrounds.

## Discussion

CD4^+^ T cells play a central role in the defense against TB associated with adaptive immune mechanisms. Investigating the molecular immune mechanisms in the CD4^+^ T regulatory TB process contributes to efforts to individualize TB prevention and management. Herein, we employed integrated bioinformatics approaches to construct a ceRNA network. More importantly, we demonstrated the relationship between this ceRNA network and some CD4^+^ T subsets. This study was performed based on large-scale and widely accepted TB datasets, thus improving the credibility and representation of the present study. With the coronavirus disease 2019 (COVID-19) pandemic, the relationships between COVID-19 and TB have drawn increasing attention from the immune community. The influence of CD4^+^ T subsets on TB and COVID-19 is complex and multidimensional (Yang and Lu, 2020). Our results shed new light on further investigation into the mechanism of COVID-19 and other respiratory infectious diseases.

The limitations of the data mining strategy in this study were also apparent in the number of various T cells that were not directly accessible. Thus, we used the ImmuCellAI platform to estimate the abundance of T cells based on the gene set signature method. However, the most practical experience of this platform was obtained from oncology studies. We calculated the abundance of peripheral blood T cells in TB cases and observed lower levels of CD4^+^ T, Tr1, nTreg, iTreg, and Tfh in active cases. Some of these results were supported by circumstantial evidence. The present study performed an essential attempt to use ImmuCellAI in its investigation of TB immunological mechanisms, the results of which provided a theoretical reference for the analysis of the molecular mechanisms involved in the interactions between pathogens and the immune system.

The protective role of CD4^+^ T is among the most critical for inhibiting TB activity, with the depletion of CD4^+^ T promoting TB progression and increasing the risk of death (Foreman et al., 2016; Santos-Pereira et al., 2021). The number of polyfunctional *M. tb* antigen-specific CD4^+^ T cells in the peripheral blood demonstrated an inverse relationship with the pathogen burden in the lungs (Day et al., 2011). Tfh is an important CD4^+^ T-cell subset that supports humoral immunity and facilitates neutralizing antibody responses to control pathogens (Crotty, 2019). Although the mechanisms remain unknown, a decrease in peripheral blood Tfh levels in active cases compared to those in latent cases has been reported (Kumar et al., 2014). However, the Tregs estimations in this study were controversial. Previous small-scale studies (Semple et al., 2013; Stringari et al., 2021) suggested an increasing trend of peripheral blood Tregs counts in active TB cases compared to the numbers in latent cases. Due to the methodological limitations of the studies, the abundance of Tregs mainly represented cell activity but not necessarily the exact count. As supporting evidence, the functional enrichment results of genes positively related to Tregs indicated that the decrease of those genes in active TB probably suppressed the activation processes of Tregs rather than proliferation. Tregs attenuate inflammation during chronic infectious disease, which maintains immune homeostasis against host damage from excessive inflammation. However, the adverse effect was that pathogen clearance was suppressed (Stephen-Victor et al., 2017). Tregs play a complex role during *M. tb* infection; in different stages of TB progression, Tregs are redistributed between the peripheral blood and local inflammatory sites; thus, their regulatory effect might be dual (Boer et al., 2015). The regulatory details of various Treg sub-types in TB progression were not previously elucidated. Our results demonstrated the potential possibilities of Treg-related mechanisms in TB activation and called for larger-scale investigations with precise designs.

The major cluster (cluster I) genes regulated by the ceRNA network were associated with T-cell behaviors in biological processes and pathways. The enriched genes were down-regulated in active cases, which indicated their suppressed activation, differentiation, and development during TB activity, echoing the estimation of T-cell subset abundance in the present study. We found that the PD-1 checkpoint was one of the critical pathways in TB activation. PD-1 inhibitors are widely used in patients with tumors. Latent TB reactivation, as a severe adverse event, has attracted much attention (Picchi et al., 2018; Anand et al., 2020). The PD-1 pathway plays a critical role in various infectious diseases (Sharpe et al., 2007). *M. tb* infection elevates PD-1 expression in peripheral blood CD4^+^ T cells, which likely limits the host immune response against pathogens (Shen et al., 2016). PD-1 inhibition promotes TNF-α secretion and results in increased *M. tb* growth. Consistent with this, the peripheral blood expression of CD4^+^ T PD-1 declines (Tezera et al., 2020). However, the mechanisms by which the PD-1 pathway regulates TB progression remain unknown. Therefore, our findings provide novel insight for further exploration.

This study constructed a ceRNA network mediated by the MIR600HG/hsa-mir-21-5p axis and included five targeting mRNAs (BCL11B, ETS1, EPHA4, KLF12, and KMT2A). A few studies have demonstrated the function of MIR600HG, which is considered a lncRNA biomarker for predicting the progression of tumor patients (Song et al., 2018; Cao et al., 2022). Xiao et al. (2021) reported that MIR600HG, which was involved in the ceRNA network in the present study, influenced pancreatic adenocarcinoma progression by regulating immune cell infiltration. The present study investigated the regulatory role of MIR600HG in infectious diseases. Our results demonstrated the great promise of MIR600HG in immunology. hsa-mir-21-5p is considered a hot-spot miRNA in immune research associated with TB (Kozomara et al., 2019). However, opposing views debate the expression difference in active cases. Kleinsteuber et al. (2013) reported decreased hsa-mir-21 levels in active cases compared to those in latent cases. However, two independent studies reported higher hsa-mir-21 expression in active cases compared to that in healthy controls and patients receiving anti-TB treatment (Wang et al., 2018; Kathirvel et al., 2020). Previous studies revealed that hsa-mir-21-5p mitigated inflammatory responses by regulating macrophages (Zhao et al., 2019). Carissimi et al. (2014) identified hsa-mir-21 as a negative modulator of T cells and the T-cell receptor (TCR) as the critical pathway. Nguyen et al. (2021) confirmed that hsa-mir-21 regulated the cellular functions and apoptosis of CD4^+^ T through the TCR pathway in infectious diseases. The results of the present study showed revealed an hsa-mir-21-5p regulatory network presumably linked to the TCR regulating CD4^+^ T subsets during TB activation, which supplemented our understanding of the role of hsa-mir-21-5p in TB progression.

BCL11B, ETS1, EPHA4, KLF12, and KMT2A were down-regulated in active cases. ROC analysis results confirmed the diagnostic prospects. Most of these findings are consistent with those previous independent reports (Jezela-Stanek et al., 2020; Zhao et al., 2020; De Araujo et al., 2021; Natarajan et al., 2022). We also confirmed their accuracy in distinguishing active TB and, more importantly, explored the ceRNA regulatory mechanism and affected T-cell types. Complementarity prediction and expression correlated analysis revealed the potential role of the MIR600HG/hsa-mir-21-5p axis in mediating these genes. The integrated results of the co-expression analysis, PPI network construction, and enrichment indicated that the critical CD4^+^ T subsets likely influenced TB progression *via* a functional gene cluster containing these five genes. BCL11B and ETS1 play key roles in the development of CD4^+^ T subsets (Liu et al., 2010; Garrett-Sinha, 2013); as transcription factors, they are deeply involved in Treg and Tfh activation and differentiation (Li et al., 2010; Kitagawa et al., 2017; Kim et al., 2018). EPHA4, as a receptor tyrosine kinase, has been implicated in the mediation of cell developmental events and is associated with the maturation and development of CD4^+^ T cells (Munoz et al., 2006). KLF12 and KMT2A are gene expression-regulated transcription factors associated with T cell proliferation (Peterson et al., 2018; Parrado, 2020). The experimental evidence presented above theoretically supported our hypothesis, which proposed that decreased levels of these five mRNA biomarkers suppress T-cell activity in active TB cases.

Compared to previous data-driven studies (Sweeney et al., 2016; Singhania et al., 2018; Natarajan et al., 2022), the present study was driven by immunological mechanisms to screen for gene biomarkers. In practice, this dual strategy approach provides more useful information. The elastic net regression model was used to determine the coefficients of each feature to obtain an integrated model to assess the compounded accuracy. For revealing the mechanisms, despite sacrificing some diagnostic accuracy, our elastic net scoring classifier still showed excellent performance in distinguishing active cases. The validation results in the testing set confirmed that our findings were robust and that the classifier model was fitted appropriately. As demonstrated in this study, the MIR600HG/hsa-mir-21-5p axis ceRNA network regulated TB activation *via* some subsets of CD4^+^ T cells; thus, the performance of our findings in CD4^+^ T-cell-deficient populations should be assessed. In the real-world TB disease spectra, cases with HIV coinfection are probably the most representative samples with CD4^+^ T deficiency. The cases also have a higher prevalence of hospitalization and mortality compared to HIV-negative cases (Bruchfeld et al., 2015; Subbarao et al., 2015). The HIV-positive internal validation confirmed the adequate diagnostic accuracy of our hypothesis in CD4^+^ T-cell-deficient cases. Further in-depth exploration of the effect of HIV on TB activity was not within the scope of the current study. However, the five critical T-cell subsets and several gene biomarkers identified in the present study are also reportedly affected by HIV infection (Jasenosky et al., 2015; Zhou et al., 2021). The results of the present study also suggest implications for future research on the molecular mechanisms by which HIV facilitates TB activity. Our findings were based on an adult cohort; however, children living with TB deserve special attention (Marais et al., 2014). Constrained by the target population size, we were unable to enroll a new cohort; however, we performed validation in comparable high-quality datasets. The results suggested that our findings were also valid for pediatric cases. It is worth noting that the peripheral T-cell compartment could change with aging (Arsenovic-Ranin et al., 2017; Reynaldi et al., 2019). As shown in Supplementary Table S7, the diagnostic accuracy of nTreg in child cases was low. The immune effect of nTreg on tuberculosis may be affected by aging factors (Namdeo et al., 2020). The issue is worthy of further exploration.

## Conclusion

The results of this study identified a T-cell-related MIR600HG/hsa-mir-21-5p axis ceRNA network, which likely revealed the immunological mechanisms associated with TB activation. The results of the internal and external validations confirmed that our findings applied to various populations with different backgrounds. Although some results remain controversial, we believe that this ceRNA network helps uncover the CD4^+^ T subsets associated with the regulatory mechanisms in TB activation and provides prospects for clinical applications.

## Data Availability

The datasets presented in this study can be found in online repositories. The names of the repository/repositories and accession number(s) can be found in the article/Supplementary Material.

## Supplementary Material

Supplementary Figure S1 Volcano diagram of DEGs in training. Red dots represented up-regulated genes in active TB cases, blue dots represented down-regulated genes.

Supplementary Figure S2 Comparision of critical T cells abundance between active and latent TB cases in testing set by t-test. * *p* < 0.05, ** *p* < 0.01, *** *p* < 0.001, and **** *p* < 0.0001.

Supplementary Figure S3 Comparision of critical T cells abundance between active and latent TB cases in HIV negative/positive sub-cohorts and external cohort by t-test. * *p* < 0.05, ** *p* < 0.01, *** *p* < 0.001, and **** *p* < 0.0001. **(A)** HIV negative sub-cohort. **(B)** HIV positive sub-cohort. **(C)** external cohort.
