# Supplementary figures and images for: Identification and validation of a T-cell-related MIR600HG/hsa-mir-21-5p competing endogenous RNA network in tuberculosis activation based on integrated bioinformatics approaches
Source: Front Genet. 2022 Sep 20;13:979213. doi: 10.3389/fgene.2022.979213 (PMC9531151; doi:10.3389/fgene.2022.979213)

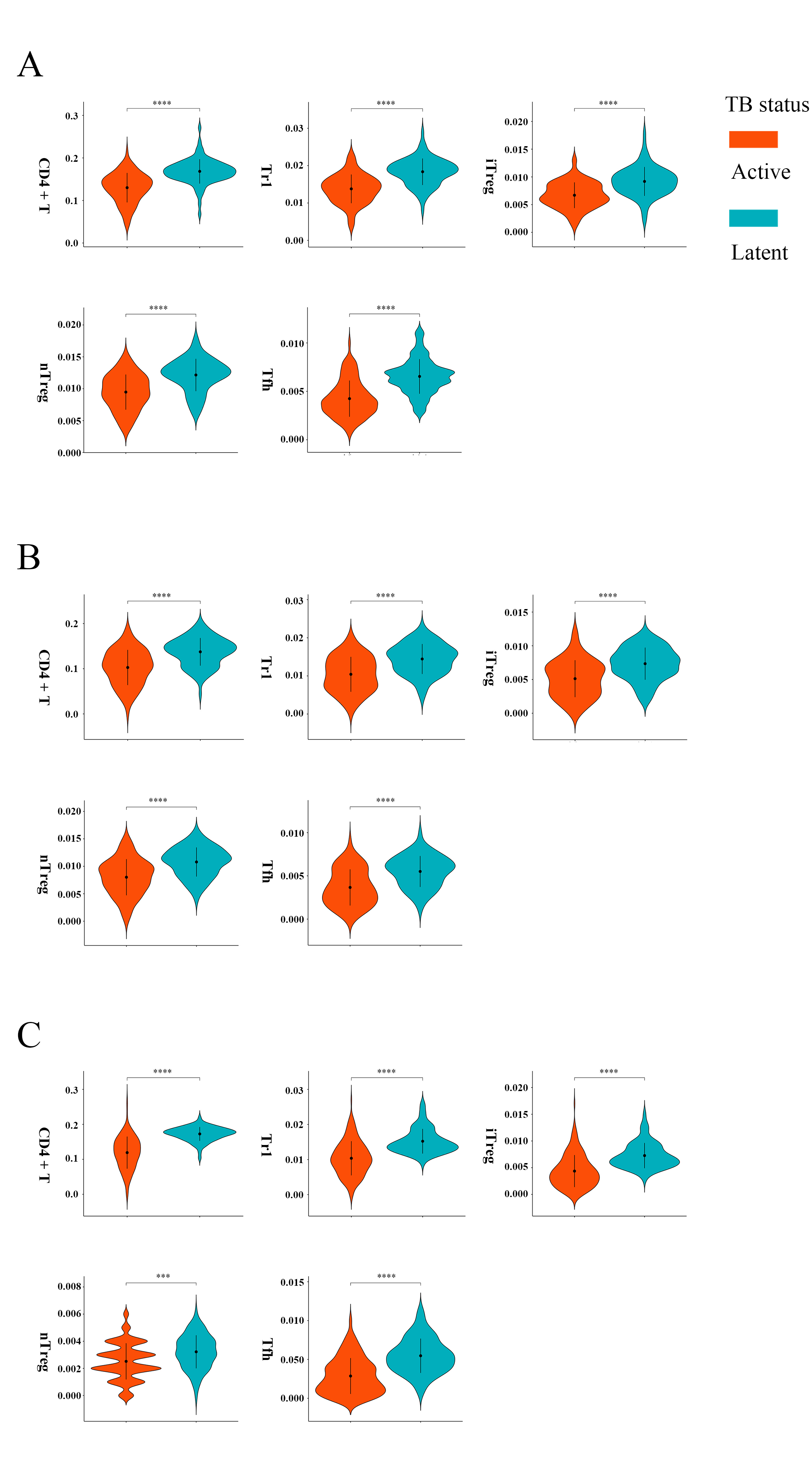

Supplement: Supplementary file 3 [file Image3.TIF]

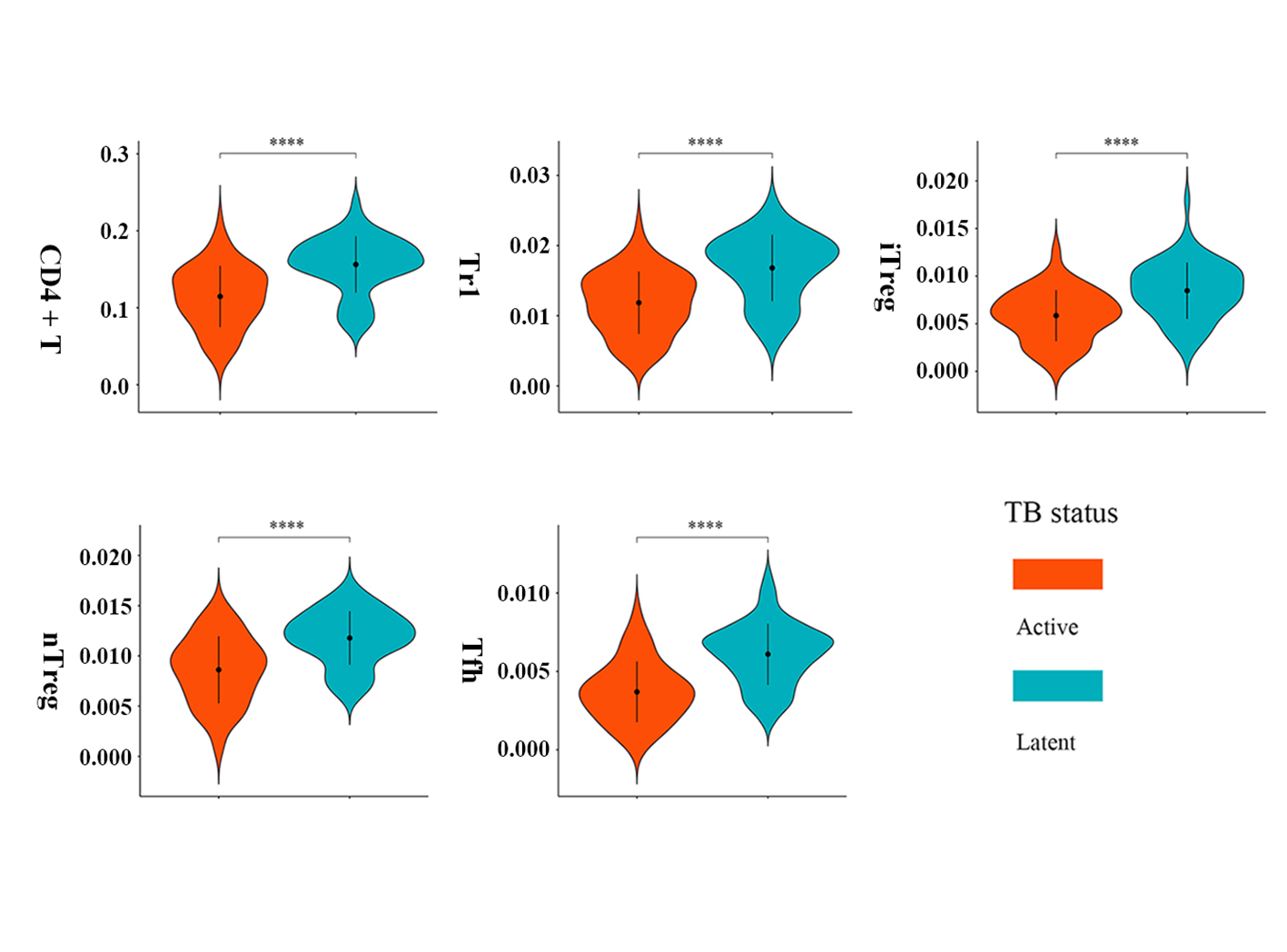

Supplement: Supplementary file 4 [file Image2.TIF]

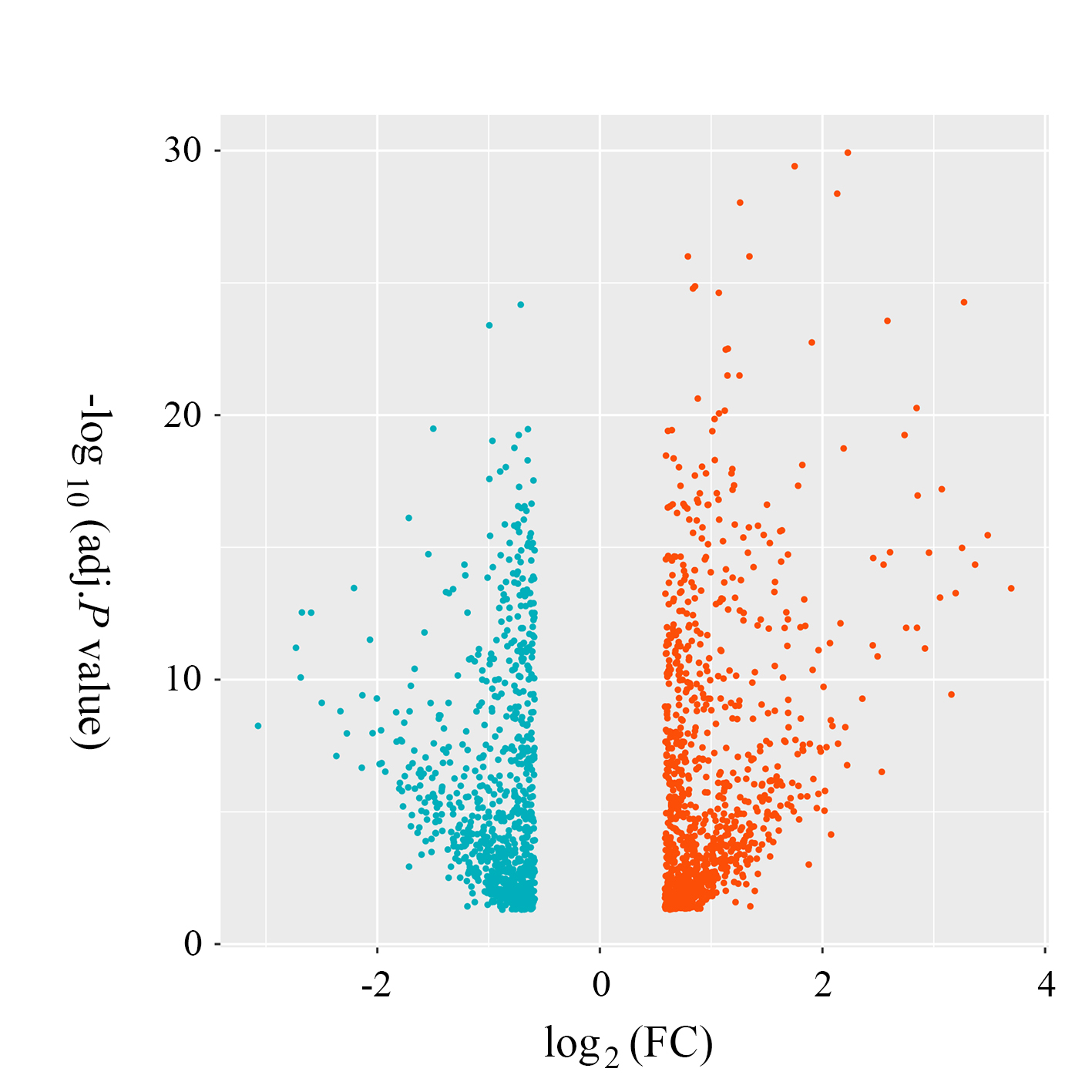

Supplement: Supplementary file 5 [file Image1.TIF]
